# Academy of Medicine, Paris

**Published:** 1888-11

**Authors:** 


### *ACADEMY OF MEDICINE, PARIS.*

[From *Le Progrès Médical.*]

Translated by Dr. A. DAGENAIS, Buffalo.

### Meeting of October 2, 1888.

M. Hérard, President, in the chair.

M. Verrier read a communication on a case of *enchâtonnement* of the placenta with septicemia, and where a cure was affected by antiseptic injections. The *enchâtonnement* took place towards the right cornu of the uterus, abnormally dilated.

Injections were made with a solution of sublimate 1–2000, with the double current sound of Budin. On the ninth day the septicemic phenomena were increasing. Injections of carbolic acid, 2.5 per cent., were then used. Twenty-one days after confinement the patient, forty years of age, multipara, had completely recovered.

This observation goes to prove that curetting and swabbing are not indispensables in serious cases, and that antiseptic injections can, by themselves, effect a cure.

M. Bouchard read for M. Mossé (of Montpelier) a communication on "Glycosuria and Polyuria in Paludisme." The urologia of the "palustre-fever" deserve to be taken up again. The classical propositions on the character and the course of the urologic syndroma during the paroxysms, and after the access offers a great many exceptions, it is only with reserve that it may be given as a rule. After an access of intermittent fever, glycosuria may appear, but it is a rare fact, and generally transitory; and glycosuria, which appears under these circumstances, seems to depend upon inherent conditions of the individual, or to be the result of a special state of the organism, whether transient or permanent (arthritic diathesis, lactation, etc.).

The telluric intoxication has in its production only one secondary action, for, with the cachectics *paludeêns* and in the intermittent pernicious fever, glycosuria does not seem to be more frequent than in *paludisme*, though less serious or more inveterated. After the access of intermittent fever, it is frequent, but not constant, to see an acute or transient polyuria appear. The quantity of urine varies from two litres and a-half to three and one-half in the twenty-four hours; it has even reached five or eight. This acute polyuria begins, usually, several days after the access. It is not accompanied by a proportional azoturia. We must not look upon it as a simple hydruria.

The chlorides in particular, where they had been given, were found in great quantity. By its manner and principles of character, this polyuria is nearer to polyuria observed in the convalescence of acute disease. Instead of constituting really a critical syndroma to know exactly its nature, it would be well to ascertain whether or not this polyuria is accompanied by an increase of urinary toxicity. From a semiologic point of view, we must recommend that, during several consecutive days, an examination of the urine should be made after the disappearance of the febrile accesses.

The fact observed by S. Ringer, that the sulphate of quinine would dissolve the elements of the fever, deserves to be confirmed by new analyses.

M. Laborde read a communication on "Alcohol and its toxicity, on alcohol so-called refined and commercial, and on artificial bouquets."

Commerce, with the help of chemistry, adds to natural products some artificial products, with which she strives to give the intrinsic qualities of these natural products. These artificial products constitute, in reality, dangerous poisons. Wine is made of all sorts, mixed with alcohol, some coloring matter, and one essential oil, to which the name of bouquet is given. The bouquet is a complex product; there are two varieties, the oil of the French wine, and the German.

These bouquets may determine a toxical accident. The alcohol is a convulsive poison; it derives its convulsive power from two aldehydes: the pyromucic aldehyde, or furfurol, and the salicylic aldehyde. The convulsive and epileptic faculty of the furfurol is produced only after being injected into the veins, and not where introduced into the stomach. The salicylic aldehyde and the salicylate of methyl give to these liquors and bouquets this severe convulsive power. Among these bouquets some are inoffensive, but most of them are poisonous. The type of these poisons is contained in absinthe. In the vermouth, and the bitters, there is also one of the most dangerous artificial bouquets, the salicylic aldehyde. This aldehyde, injected into the veins of animals, determined epileptiform attacks. The vermouth and the bitter may also contain a convulsive substance, the salicylate of methyl.

M. Dujardin-Beaumetz estimated that it would be wrong to conclude from the experiences of M. Labordes to the clinical reality. M. Laborde injected these toxic substances into the veins, from which immediate alterations of the blood took place, and the possible formation of embolism. But these same substances, introduced into the stomach, sustain modifications before passing into the blood, and would not give rise to the accidents described by Mr. Laborde. What do the clinical facts establish? It is demonstrated that the absinthics have epileptic attacks absolutely like the alcoholics. This accident does not result from intoxication, but from varied degenerations of the marrow.

M. Duplay read a report on the Meyuot prize for 1888.

